# Draft Genome Sequences of Bacteroides pyogenes Strains Isolated from the Uterus of Holstein Dairy Cows with Metritis

**DOI:** 10.1128/MRA.01043-19

**Published:** 2019-10-10

**Authors:** Federico Cunha, Soo Jin Jeon, KwangCheol Casey Jeong, Klibs N. Galvão

**Affiliations:** a Department of Large Animal Clinical Sciences, College of Veterinary Medicine, University of Florida, Gainesville, Florida, USA; b Department of Animal Sciences, University of Florida, Gainesville, Florida, USA; c Emerging Pathogens Institute, University of Florida, Gainesville, Florida, USA; d D. H. Barron Reproductive and Perinatal Biology Research Program, University of Florida, Gainesville, Florida, USA; University of Arizona

## Abstract

Bacteroides pyogenes is found in the human and animal gut and is implicated in the pathogenesis of metritis in cows. We report the draft genome sequences of four Bacteroides pyogenes isolates obtained from the uterus of metritic cows. This will increase the understanding of its pathogenicity, antimicrobial resistance, and differentiation across hosts.

## ANNOUNCEMENT

Bacteroides pyogenes is an obligate anaerobic, nonmotile, non-spore-forming, Gram-negative bacillus found in human and animal gut microbiota and has pathogenic capabilities (1–3). The bacterium was first isolated from swine feces and abscesses along with a purported species, Bacteroides suis, which was later reclassified as a heterotypic synonym of Bacteroides pyogenes (4–6). Recent studies observed that cows with metritis have a higher abundance of Bacteroides pyogenes in their uterus than do healthy cows (3, 7). Genome analyses of Bacteroides pyogenes isolates from a cat and swine revealed a diversification of strains across hosts and a need for further genome analyses to improve our understanding of the species (8). In this study, we sequenced the genomes of four Bacteroides pyogenes strains isolated from the uterus of dairy cows at the time of metritis diagnosis.

Swab samples were taken in June 2016 at the University of Florida’s Dairy Research Unit in Hague, FL. The swabs were suspended in 1,000 μl of Luria-Bertani broth (Sigma-Aldrich). Twenty microliters of suspension was inoculated onto Wilkins-Chalgren agar with GN spore anaerobic supplement and kanamycin and incubated for 48 h at 37°C under anaerobic conditions. Colonies with light pigmentation were subcultured on Wilkins-Chalgren anaerobe agar with GN spore anaerobic supplement. Sanger sequencing of the 16S rRNA gene of each isolate was performed by Genewiz (South Plainfield, NJ). BLAST comparisons were performed against the NCBI nucleotide collection, and those with an identity of ≥99% were confirmed to be Bacteroides pyogenes (9). The confirmed Bacteroides pyogenes strains were named KG-29, KG-30, KG-31, and KG-32.

After initial isolation, each of the four strains was cultivated a single time for bulk growth on Wilkins-Chalgren anaerobe agar with Gn spore anaerobic supplement for 48 h at 37°C under anaerobic conditions and harvested for genomic DNA (gDNA) extraction. Extraction of gDNA was done with the DNeasy blood and tissue kit (Qiagen) according to the manufacturer’s instructions for Gram-negative bacteria. The Nextera XT kit (Illumina, Inc.) was used for library preparation, following the manufacturer’s instructions, and it was loaded into the MiSeq reagent kit V2. Sequencing was performed on the MiSeq platform (Illumina, Inc.) with a 2 × 250-bp 500-cycle cartridge. KG-29 had 1,211,489 reads with a genome coverage of 86×. KG-30 had 1,232,697 reads with a genome coverage of 88×. KG-31 had 1,111,352 reads with a genome coverage of 79×. KG-32 had 1,303,278 reads with a genome coverage of 93×. Sickle version 1.33.1 (10) was used to trim FastQ data using a quality parameter of 30 and length of 50. k-mer values of 21, 33, 55, 77, 99, and 127 were used for *de novo* assembly on SPAdes version 3.11.1 (11). PGAP (12) and PATRIC (13) were used to annotate the assembled genomes. All software was used with default parameters, unless otherwise specified. The genomic features of the four isolates are displayed in Table 1.

**TABLE 1:** Genome statistics and features of Bacteroides pyogenes strains

Isolate	No. of reads	Coverage (×)	Length (bp)	No. of contigs	*N*_50_ (bp)	GC content (%)	No. of CDSs^*a*^	No. of rRNAs	No. of tRNAs
KG29	1,211,489	86	3,373,258	124	96,943	45.98	3,251	8	60
KG30	1,232,697	88	3,536,156	176	76,873	45.72	3,414	7	60
KG31	1,111,352	79	3,542,408	155	99,174	45.79	3,468	8	60
KG32	1,303,278	93	3,260,535	177	50,766	45.88	3,087	4	60

a CDSs, coding sequences.

The presence of antibiotic resistance genes (ARGs) was varied across the strains, with a beta-lactam resistance gene (*cfxA2*) being the only ARG shared by all four strains (14). KG-32 is the only strain lacking tetracycline resistance genes, while KG-29, KG-30, and KG-31 had 5, 1, and 7 copies of a tetracycline ribosomal protection gene (*tetQ*), respectively (15). KG-29, KG-31, and KG-32 contain a dihydropteroate synthase type-2 gene (*sul2*) which can confer resistance to sulfonamides (16). These Bacteroides pyogenes genomes obtained from cow hosts will enable a better understanding of the species’ differentiation across host species.

### Data availability.

These whole-genome sequences are available at DDBJ/EMBL/GenBank under accession numbers VKLY00000000 (KG-29), VKLX00000000 (KG-30), VKLW00000000 (KG-31), and VKLV00000000 (KG-32). This project and the trimmed reads have been uploaded into the NCBI Sequence Read Archive under BioProject number PRJNA553588 and accession numbers SRS5092517 (KG-29), SRS5092516 (KG-30), SRS5092515 (KG-31), and SRS5092514 (KG-32).
